# Obesity and Alzheimer's disease, does the obesity paradox really exist? A magnetic resonance imaging study

**DOI:** 10.18632/oncotarget.26162

**Published:** 2018-10-05

**Authors:** Jordi Pegueroles, Amanda Jiménez, Eduard Vilaplana, Victor Montal, María Carmona-Iragui, Adriana Pané, Daniel Alcolea, Laura Videla, Anna Casajoana, Jordi Clarimón, Emilio Ortega, Josep Vidal, Rafael Blesa, Alberto Lleó, Juan Fortea, for the Alzheimer's Disease Neuroimaging Initiative

**Affiliations:** ^1^ Memory Unit, Department of Neurology, Hospital de la Santa Creu i Sant Pau-Biomedical Research Institute Sant Pau, Barcelona, Spain; ^2^ Centro de Investigación Biomédica en Red de Enfermedades Neurodegenerativas (CIBERNED), Madrid, Spain; ^3^ Obesity Unit, Department of Endocrinology and Nutrition, Hospital Clinic Universitari de Barcelona, Barcelona, Spain; ^4^ Department of Gastrointestinal and Obesity Surgery, Hospital de Barcelona-SCIAS, Barcelona, Spain; ^5^ Institut d'Investigacions Biomèdiques August Pi Sunyer (IDIBAPS), Barcelona, Spain; ^6^ Centro de Investigación Biomédica en Red de la Fisiopatología de la Obesidad y Nutrición (CIBEROBN), Barcelona, Spain; ^7^ Centro de Investigación Biomédica en Red de Diabetes y Enfermedades Metabólicas asociadas (CIBERDEM), Barcelona, Spain

**Keywords:** preclinical Alzheimer's disease, obesity, weight loss, magnetic resonance imaging, body mass index

## Abstract

Mid-life obesity is an established risk factor for Alzheimer's disease (AD) dementia, whereas late-life obesity has been proposed as a protective state. Weight loss, which predates cognitive decline, might explain this obesity paradox on AD risk. We aimed to assess the impact of late life obesity on brain structure taking into account weight loss as a potential confounder. We included 162 elderly controls of the Alzheimer's Disease Neuroimaging Initiative (ADNI) with available 3T MRI scan. Significant weight loss was defined as relative weight loss ≥5% between the baseline and last follow-up visit. To be able to capture weight loss, only subjects with a minimum clinical and anthropometrical follow-up of 12 months were included. Individuals were categorized into three groups according to body mass index (BMI) at baseline: normal-weight (BMI<25 Kg/m^2^), overweight (BMI 25-30 Kg/m^2^) and obese (BMI>30 Kg/m^2^). We performed both an interaction analysis between obesity and weight loss, and stratified group analyses in the weight-stable and weigh-loss groups. We found a significant interaction between BMI and weight loss affecting brain structure in widespread cortical areas. The stratified analyses showed atrophy in occipital, inferior temporal, precuneus and frontal regions in the weight stable group, but increased cortical thickness in the weight-loss group. In conclusion, our data support that weight loss negatively confounds the association between late-life obesity and brain atrophy. The obesity paradox on AD risk might be explained by reverse causation.

## INTRODUCTION

Dementia and obesity are increasing in epidemic proportions among western societies [1, 2]. The total number of people with dementia is expected to rise to 106 million worldwide by 2050 [1]. Alzheimer's disease (AD) is the main cause of dementia, but there are no current preventive or disease modifying treatments. Therefore, the identification of modifiable risk factors is of major interest.

Obesity is a well-established risk factor for type 2 diabetes (T2D), cardiovascular disease and cancer, and it has also been proposed as an independent risk factor for dementia and AD [3–5]. Mid-life obesity has been consistently identified as a risk factor for dementia and AD in epidemiological studies [3]. Moreover, several pathological alterations associated with obesity, such as insulin resistance, inflammation or mitochondrial dysfunction, have also been related to AD pathological processes [6]. Finally, mid-life obesity has been linked to greater burden of AD neuropathology in the human brain [7]. However, the association between late-life obesity and dementia is inconclusive [8–11]. Some studies have even identified late-life obesity as a protective state for dementia [10], hence the “obesity paradox” on AD risk.

An “obesity paradox” has been described in relation to other health conditions [12, 13], including cardiovascular disease [13], and might be explained by bias [14]. Stokes et al. nicely demonstrated that the “obesity paradox” on cardiovascular mortality is mainly the result of reverse causation, produced by the confounding effect of illness-related weight loss [15]. In this sense, the relationship between dementia and weight loss is widely recognized [16]. More recently, weight loss has been related to preclinical and prodromal AD stages up to 20 years prior to symptom onset [17–19]. This long period of weight loss in preclinical AD might obscure the relationship between late-life obesity and AD, and might explain the “obesity paradox” on AD risk.

Structural MRI has been extensively used to study aging and AD [17, 20–22]. Several studies have evaluated the relationship between obesity and brain structure, with conflicting results [23–36]. Conversely, unintentional weight loss is consistently associated with both cross sectional and longitudinal brain atrophy in healthy elderly [26, 31, 35, 37].

In the present study, we aimed to assess the brain structural changes associated with obesity in healthy elderly from the Alzheimer's Disease Neuroimaging Initiative (ADNI), taking into account the potential confounding effect of weight loss.

## RESULTS

Table 1 summarizes the demographic, biometric and biochemical data according to BMI at baseline. We included 162 subjects (80 female) in the study, with a mean age of 73.3±6.0 years (ranging between 56.2 to 89.0 years) and a mean BMI of 27.4±4.0 Kg/m^2^. Fifty-two were in the normal-weight category (32.1%), 70 in the overweight category (43.2%) and 40 in the obese category (24.7%). None of the study participants presented a BMI below 20.0 Kg/m^2^. Triglycerides levels were higher in obese subjects respect the other two groups and in overweight subjects respect normal-weight subjects, but there were no other significant differences. In the follow-up, 36 subjects had a significant weight loss (≥5%). No significant differences in age, sex, BMI, baseline cognitive performance or APOE genotype were observed between those subjects with or without weight loss.

**Table 1:** Demographic, anthropometric and neuropsychological data according to the weight categories

	Normal-weight	Overweight	Obese	p
n	52	70	40	
Gender (females), N (%)	24 (46.2)	41 (58.6)	15 (37.5)	0.089
Age (years)	74.45 (6.07)	73.15 (6.31)	72.08 (4.93)	0.159
Weight-Loss group, N (%)	15 (28.8)	11 (15.7)	10 (25.0)	0.200
SBP (mg/dL)	131.85 (17.57)	133.01 (14.67)	135.57 (13.14)	0.504
DBP (mg/dL)	73.46 (11.38)	73.89 (9.72)	76.03 (8.99)	0.443
FPG (mg/dL)	98.21 (21.70)	96.26 (13.95)	103.08 (20.97)	0.179
Cholesterol (mg/dL)	194.60 (36.91)	188.37 (38.34)	193.72 (37.43)	0.617
Triglycerides (mg/dL)	113.35 (69.68)	145.81 (75.37)	161.62 (88.68)	0.009
Education (years)	16.63 (2.63)	16.50 (2.35)	16.50 (2.46)	0.949
ApoE4 carrier, N (%)	16 (30.8)	23 (32.9)	9 (22.5)	0.507
MMSE score	29.23 (1.10)	29.00 (1.15)	29.27 (0.93)	0.344
ADAS-Cog 11 score	5.69 (2.88)	5.91 (3.45)	5.36 (2.42)	0.659
ADAS-Cog 13 score	8.82 (4.38)	9.28 (5.08)	8.16 (3.40)	0.453
Type 2 Diabetes, N (%)	4 (7.7)	5 (7.1)	6 (15.0)	0.351

Data is expressed as mean (standard deviation). Abbreviations: BMI: body mass index; SBP: systolic blood pressure; DBP: diastolic blood pressure; FPG: fasting plasmatic glucose; MMSE: Minimental score examination; ADAS-Cog Alzheimer's Disease Scale Assessment.

### The association of obesity and brain structure is confounded by weight loss

**Figure 1:**
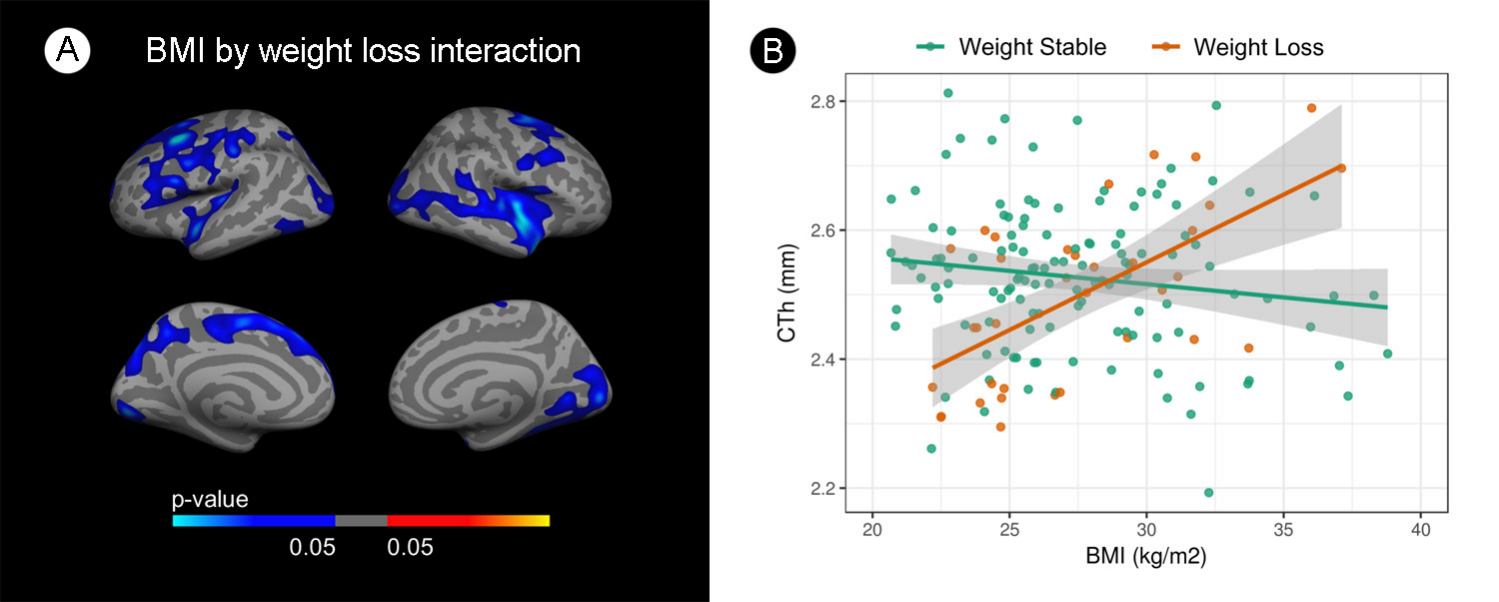
Interaction analysis between body mass index and cortical thickness by weight loss **(A)** Vertex wise analysis. Blue areas indicate regions with significant (FWE<0.05) interaction term. **(B)** Scatterplot showing this interaction. BMI is shown in the X-axes and mean cortical thickness in the y-axes at the insular-temporal cluster of the right hemisphere. Green dots represent weight-stable subjects and orange dots represent weight-loss subjects. The blue gradient color represent the uncorrected p-values (p<0.05) in the FWE surviving clusters only.

Figure 1 presents the vertexwise interaction analysis across the whole cortical mantle between BMI and weight loss. Extensive clusters emerged in both hemispheres (Figure 1A). As hypothesized, cortical thinning in relation with BMI occurred only in weight-stable subjects whereas in the weight-loss group increasing BMI was associated with increased cortical thickness. Figure 1B shows the mean CTh values for each subject at the cluster of maximum significance of the interaction term in the insular-temporal cluster of the right hemisphere. Similar results were found in all FWE corrected clusters (results not shown). The interaction analysis on HVa was not significant (p=0.4).

**Figure 2:**
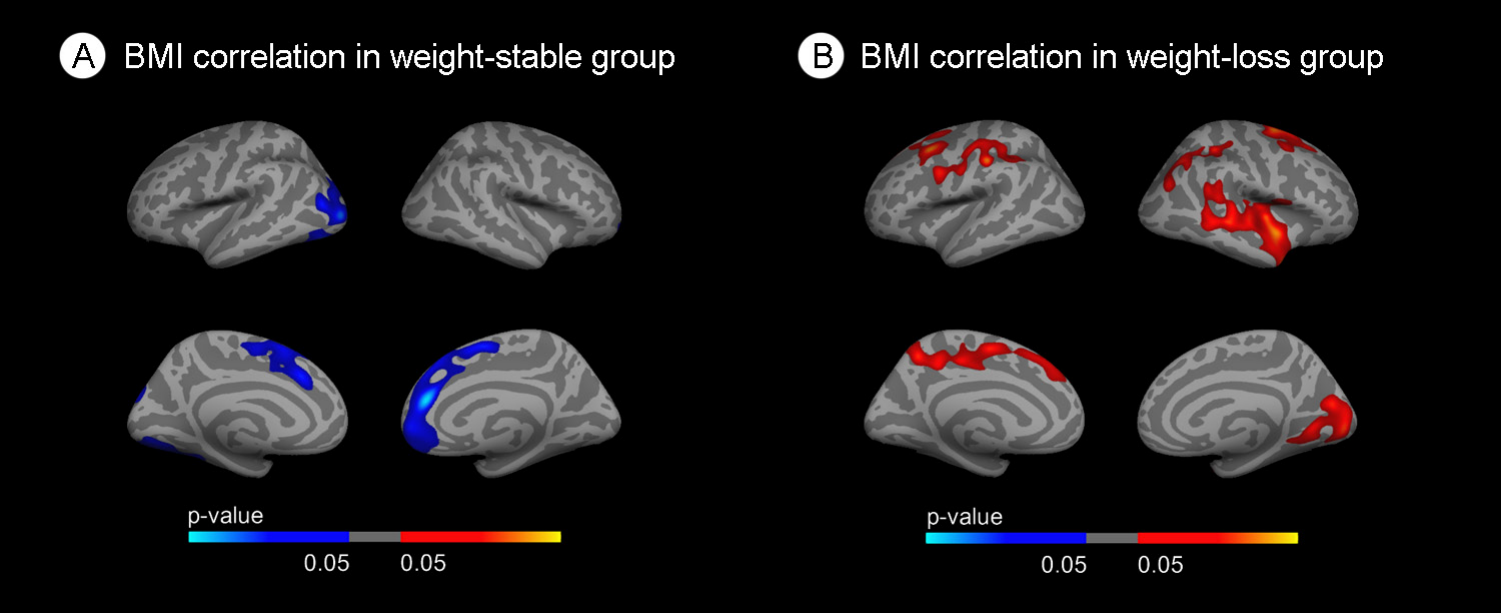
Stratified correlation analysis between body mass index and cortical thickness **(A)** Correlation between BMI and cortical thickness in the weight-stable group. **(B)** Correlation between BMI and cortical thickness in the weight-loss group. Blue areas indicate regions with significant (FWE<0.05) cortical thinning. Red areas represent areas with significant cortical thickening (FWE<0.05). The blue and red gradient colors represent the uncorrected p-values (p<0.05) in the FWE suriving clusters only.

We performed stratified vertexwise correlation analyses to further assess whether the relationship between obesity and CTh is modified by weight-loss status (Figure 2). In the weight-stable group, areas of cortical thinning with increasing BMI emerged in both hemispheres, including occipital, inferior temporal and frontal regions (Figure 2B). Conversely, in the weight-loss group, areas of cortical thickening appeared in the superior parietal and frontal regions in both hemispheres and temporal regions in the right hemisphere (Figure 2C). Of note, the association between BMI and brain structure in the whole cohort showed a weak inverse association between BMI and cortical thickness in the inferior temporal region (results not shown).

### Obesity is associated with brain atrophy in the absence of weight loss, but is a marker of brain health in the weight-loss group

**Figure 3:**
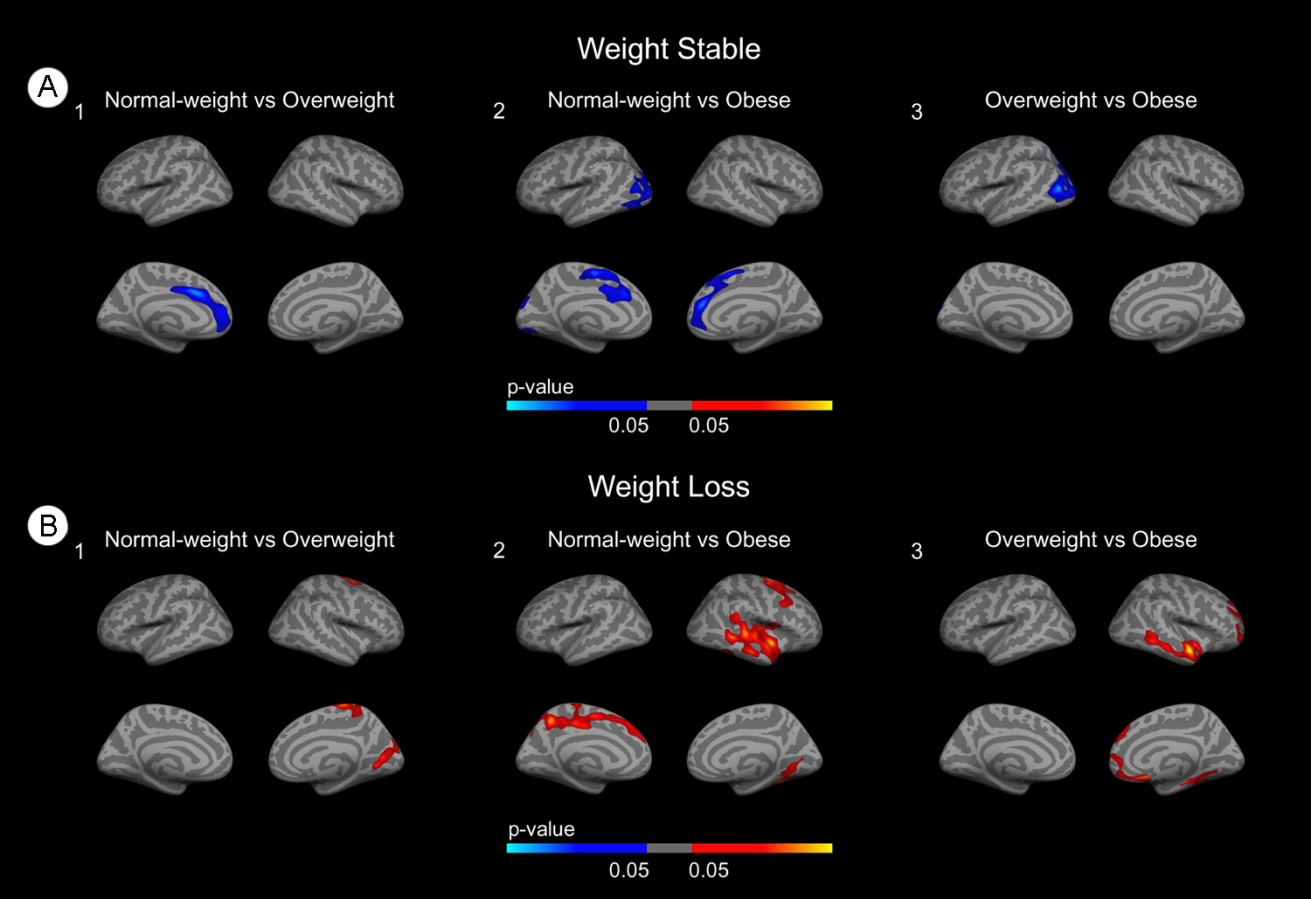
Stratified group comparisons between BMI groups **(A)** Group comparisons in the weight-stable group. **(B)** Group comparisons in the weight-loss group. Blue areas indicate regions with significant (FWE<0.05) cortical thinning. Red areas represent areas with significant cortical thickening (FWE<0.05). The blue and red gradient colors represent the uncorrected p-values (p<0.05) in the FWE surviving clusters only.

To separatedly assess the impact of obesity in the weight-stable and weight-loss groups we performed BMI group comparisons stratifying by weight-loss. We first analyzed the weight-stable group. In the overweight vs. obese comparison (Figure 3A1) clusters of decreased CTh emerged in frontal areas. In the normal-weight vs obese comparison clusters of decreased CTh in the obese subjects emerged in frontal and occipital regions of both hemispheres (Figure 3A2). Finally, in the overweight vs obese comparison (Figure 3A3), clusters of decreased CTh emerged in occipital regions. There were no significant differences in HVa between the BMI groups in the weight-stable group (p = 0.3).

We next analyzed the weight loss group. In the normal-weight vs overweight and overweight vs obese comparisons (Figures 3B1 and 3B3) we found clusters of increased CTh in temporal and posterior regions. In the normal-weight vs obese comparison (Figure 3B2) widespread clusters of increased CTh emerged. Of note, in the BMI group comparisons in the whole cohort, overweight participants showed several clusters of increased CTh when compared both with the normal-weight group and with the obese group, but no significant differences were found in the normal-weight vs. obese comparison (results not shown). There were no significant differences in HVa between the BMI groups in the weight-loss group (p = 0.09).

## DISCUSSION

In this study, we examined the relationship between late-life obesity and brain structure in healthy elderly taking into account the potential confounding effect of weight loss. Our results suggest that late-life obesity is associated with cortical thinning, but that this relationship is negatively confounded by weight-loss.

Weight loss modifies the effect of late-life obesity on brain structure supporting the hypothesis of reverse causation (the negative confounding effect of AD related weight loss) as an explanation for the obesity paradox on AD risk. We found an inverse association between BMI and CTh in the weight-stable group, but an inverse relationship in the weight-loss group suggesting that obesity promotes cortical atrophy in the absence of weight loss, but could be a marker of brain health in later stages of the disease when weight-loss modifies body composition and more degeneration has occurred. These results are thus in agreement with the concept of mid-life obesity as an established risk factor for AD [3, 7], and as a risk factor for longitudinal cortical thinning in mid-life as shown in a study with 405 middle-aged subjects (mean age 47.2 years) [31, 38]. More recently, weight loss has been identified as a non-cognitive manifestation of preclinical AD [19, 37] and we have previosly shown that weight-loss is associated with cortical atrophy [37]. In short, the weight-loss associated with preclinical AD would change the relationship between BMI and CTh in a slow, but cumulative process leading to reverse causation.

Elderly subjects have a higher prevalence of preclinical AD and longer mean preclinical periods than younger participants [38]. Reverse causation would be thus more evident in elderly subjects, and might help explain the conflicting results found in the literature. In this respect, previous works in younger cohorts have found hippocampal atrophy (mean age 63.2 years) [25] and cortical thinning in the entorhinal cortex and the posterior cingulate (mean age 63 years) in relation with obesity [31]. However, works in older cohorts have found increased hippocampal volume (mean age 74.4) [34] and increased cortical thickness in the precuneus (mean age of 76 years, also using the ADNI cohort) [27]. Our hypothesis could also explain the results by Raji et al. [30]. In this study, the authors showed an inverse correlation between late-life BMI and hippocampal, frontal lobe and anterior cingulate volumes in an older cohort (mean age 77 years). This study, however, excluded those subjects that converted to dementia in the following 5 years after the MRI. The exclusion of these subjects might have minimized reverse causation and might explain the similarities with our stratified analysis. Of note, our differences were restricted to the brain cortex; we did not find significant differences in hippocampal volume related with obesity.

Our study has several limitations. First, weight trajectories previous to MRI were not recorded. Consequently, we assumed that future weight loss is part of a dynamic process that begins at late mid-life and, therefore, before baseline MRI scan. Second, intentional weight loss cannot be differentiated from unintentional weight loss in the ADNI cohort. However, since previous studies have shown that intentional weight loss in healthy obese subjects and in obese MCI patients has beneficial cognitive effects [39–41]. Third, the ADNI cohort excluded participants with a large vascular burden. This selection bias explains the similar metabolic profile across BMI categories in our study and precludes the generalization of our results to broad obese populations. Fourth, body composition analyses or physical activity were not assessed in the ADNI cohort. Finally, these results were mainly derived from a cross-sectional analysis and further longitudinal studies with longer follow-up are necessary to confirm a plausible deleterious effect of late-life obesity on brain health.

In conclusion, weight loss negatively confounds the deleterious effect of adiposity in the brain structure in elderly subjects and might explain the “obesity paradox” on AD risk.

## MATERIALS AND METHODS

### Study participants

Data used in the preparation of this article were obtained from the Alzheimer's Disease Neuroimaging Initiative (ADNI) database (http://adni.loni.usc.edu). The ADNI was launched in 2003 by the National Institute on Aging (NIA), the National Institute of Biomedical Imaging and Bioengineering (NIBIB), the Food and Drug Administration (FDA), private pharmaceutical companies and non-profit organizations, as a %60 million, 5-year public-private partnership. The primary goal of ADNI has been to test whether serial magnetic resonance imaging (MRI), positron emission tomography (PET), other biological markers, and clinical and neuropsychological assessment can be combined to measure the progression of mild cognitive impairment (MCI) and early AD. The Principal Investigator of this initiative is Michael W. Weiner, MD, VA Medical Center and University of California – San Francisco. ADNI is the result of efforts of many co-investigators from a broad range of academic institutions and private corporations, and subjects have been recruited from over 50 sites across the U.S. and Canada. More information can be found in the acknowledgements section (see also http://adni-info.org/). We selected all normal cognitive participants with available baseline cerebrospinal fluid data and 3T MRI scan in ADNI. Significant weight loss was defined as relative weight loss ≥5% between the baseline and last follow-up visit, as previously described [37]. To be able to capture weight loss, we only included subjects with a minimum clinical and anthropometrical follow-up of 12 months. Body mass index (BMI) was calculated as weight in kilograms divided by height in meters squared. Individuals were categorized into three groups according to BMI: normal-weight (BMI<25 Kg/m^2^), overweight (BMI 25-30 Kg/m^2^) and obese (BMI>30 Kg/m^2^). One subject presented with outlier BMI value (51.3 Kg/m^2^) and thus was excluded from the analyses.

### MRI analysis

The details of MRI acquisition and pre-processing are available elsewhere (http://adni-info.org/). All structural MRIs were first processed using the cross-sectional cortical reconstruction stream in Freesurfer v5.1.;(http://surfer.nmr.mgh.harvard.edu). The procedures have been described previously [20, 42]. The cortical thickness (CTh) was calculated as the distance from the grey/white matter boundary closest to the grey/CSF boundary at each vertex. Every estimated surface was checked in a slice-by-slice basis to detect possible segmentation errors. Thirty-three subjects of the 195 initially included in the study were excluded because of segmentation errors. Finally, CTh measure was smoothed using a Gaussian kernel of 15mm full-width at half maximum. Furthermore, the hippocampal volumes were extracted using Freesurfer v5.1 and the adjusted hippocampal volume (HVa) was computed [43, 44].

### Statistical methods

Group analyses for demographic and biometric data were done using R statistical software (R Core Team 2014. R: A Language and Environment for Statistical Computing, version 3.2.5. Available at: http://www.r-project.org). Comparisons between the BMI categories were made using ANCOVA with Tukey's post hoc test correction (p<0.05) for continuous variables, and the chi-square test for categorical variables.

To study the interaction between obesity and weight loss and its impact on the brain structure, we used three diferent approaches: interaction, stratified correlation analysis (primary analyses) and group comparison analysis (secondary analyses). First, we conducted a vertexwise analysis across the whole cortical mantle, showing regions with a BMI by weight-loss group interaction. To further study this relationship, we performed a vertexwise stratified correlation analyses with BMI in the weight stable and weight loss groups separately. Furthermore, we analized the interaction between BMI and weight-loss on the HVa. Finally, in a secondary analysis, we conducted group comparisons between the BMI categories. All analyses included age, sex and triglycerides levels as covariates.

We used monte-carlo simulations as implemented in Freesurfer to correct for multiple comparisons in a cluster-size basis (family-wise error [FWE] correction at p<0.05). The figures show only those results that survived FWE correction.
